# Supplementary material for: Uncertainties in land use data may have substantial effects on environmental planning recommendations: A plea for careful consideration
Source: PLoS One. 2021 Nov 24;16(11):e0260302. doi: 10.1371/journal.pone.0260302 (PMC8612552; doi:10.1371/journal.pone.0260302)
Supplement: Attachment — Submitted filename: Response to Reviewers.docx [file pone.0260302.s002.docx]

Dear Academic Editor,
Dear Dr. Forkuor

Thank you very much for your review of our manuscript, for the encouragement to write a second revision, and for the helpful advice from you and the two reviewers.

As suggested, we have developed a second revision of the manuscript according to the comments of the reviewers. Please find attached our revised manuscript and our response letter with detailed responses to all review comments.

We are confident that the revision has led to a further improvement of the manuscript quality. In case you have any questions, please do not hesitate to contact me.

Thank you very much!

Yours sincerely,

The authors

PONE-D-21-14058R1
Uncertainties in land use data may have substantial effects on environmental planning recommendations: a plea for careful consideration
PLOS ONE

Dear Dr.[name omitted for reasons of anonymity],

Thank you for submitting your manuscript to PLOS ONE. After careful consideration, we feel that it has merit but does not fully meet PLOS ONE’s publication criteria as it currently stands. Therefore, we invite you to submit a revised version of the manuscript that addresses the points raised during the review process.

The revisions required are minor. Please, make the necessary changes or provide the required clarifications. If a particular recommendation is not feasible, please provide cogent explanations as to why.

Please submit your revised manuscript by Oct 15 2021 11:59PM. If you will need more time than this to complete your revisions, please reply to this message or contact the journal office at plosone@plos.org. When you're ready to submit your revision, log on to https://www.editorialmanager.com/pone/ and select the 'Submissions Needing Revision' folder to locate your manuscript file.

Please include the following items when submitting your revised manuscript:

- A rebuttal letter that responds to each point raised by the academic editor and reviewer(s). You should upload this letter as a separate file labeled 'Response to Reviewers'.
- A marked-up copy of your manuscript that highlights changes made to the original version. You should upload this as a separate file labeled 'Revised Manuscript with Track Changes'.
- An unmarked version of your revised paper without tracked changes. You should upload this as a separate file labeled 'Manuscript'.

If you would like to make changes to your financial disclosure, please include your updated statement in your cover letter. Guidelines for resubmitting your figure files are available below the reviewer comments at the end of this letter.

If applicable, we recommend that you deposit your laboratory protocols in protocols.io to enhance the reproducibility of your results. Protocols.io assigns your protocol its own identifier (DOI) so that it can be cited independently in the future. For instructions see: <http://journals.plos.org/plosone/s/submission-guidelines#loc-laboratory-protocols>. Additionally, PLOS ONE offers an option for publishing peer-reviewed Lab Protocol articles, which describe protocols hosted on protocols.io. Read more information on sharing protocols at <https://plos.org/protocols?utm_medium=editorial-email&utm_source=authorletters&utm_campaign=protocols>.

We look forward to receiving your revised manuscript.

Kind regards,

Gerald Forkuor
Academic Editor
PLOS ONE

**Comments from the editors and reviewers and responses**

| **Editor comments** | **Responses** |
| --- | --- |
| The revisions required are minor. Please, make the necessary changes or provide the required clarifications. If a particular recommendation is not feasible, please provide cogent explanations as to why. | Thank you very much for the advice. We have done as suggested. |
| **Reviewer #1** |  |
| GENERAL COMMENTS:  The revised version of the manuscript is greatly improved in terms of clarity and provides an important message for the planning community. | Thank you for the kind words. |
| **Reviewer #2** |  |
| GENERAL COMMENTS:  The authors have made a good-faith effort to take into account my concerns. My suggestions below are mainly minor and mainly merely a matter of clarifying wording or terms that I find confusing, as unfortunately the revised paper is often a bit confusing. | Thank you for the kind words and the detailed re-read of the manuscript. |
| Specific Coments:  1. In a number of locations “topographical” is used where it appears “land cover” would be a better term (topographical means the form of the land – e.g. hill, valley, etc). See L57, L137 | Thank you for this important remark. We changed the wording to “spatial and statistical data” in the first case and to “land use / land cover” data in the second case as we think these are the best fitting options. |
| 2. L81-85 – The meaning of this is not clear – can you rephrase? | Thank you for flagging this. We have reformulated this section as follows:  “Finally, environmental planners often presume that an intuitively felt uncertainty may be present in their basic data, but that this will not relevantly affect the response measures. The logic behind this is that planners often strive for multifunctional measures which intentionally target a larger area than monofunctional measures that usually address only smaller areas, for example to safeguard a certain species. Along this line, planners generally presume that the inaccuracies of land use data will not affect the validity of recommended measures which address multiple purposes in an aggregated spatial unit. Furthermore, in case of precautionary measures, the uncertainties of proposals of future land uses seem to be much more relevant than uncertainties stemming from inaccuracies of cartographic land use data.” |
| 3. L107 – Adressees – not sure what this means – do you mean “Potential users”? | We have changed this to ‚potential users‘ |
| 4. L135 – Actuality – do you mean “currency” (as in the data is up to date)? Or something else? | Thanks. We changed the term to ‚currency‘ as kindly suggested. |
| 5. L145 – Pictures – replace with “images” | Changed as suggested |
| 6. Methods – do you explain how you handle your spatial overlay operations evaluating “consistency” where the pixel size differ? Specifically, how do you resize the pixels? | We added a section to explain how the raster data was resized. This reads as follows:  „In order to compare the two Digital Elevation Models (input data), the DEM200 was converted to a grid with a grid cell sizes of 50 m. Since the potential areas of the two model runs were also provided as grids with a grid cell size of 50 m, they could be compared without further transformations.” |
| 7. L191 onwards – in the results you refer to “After Walter” and “After Thiele” – I suggest making it clear by including those names in the text here and also explaining explicitly how your methods relate to those sources – e.g. are you following their procedures or using their output or what? | Thank you for flaggining this. We have rephrased the whole section, beginning with:  follows:  “The case study ‘National renewable energy planning’ explored impacts of using datasets of different uncertainty levels on the potentially suitable sites for renewable energies, taking into account both human and nature-conservation needs [20]. We used the data and results of two model runs, the first one was conducted by Walter et al. 2018 and the second one by Thiele et al. 2021, for an uncertainty analysis in order to quantify the influence of data resolution on identifying opportunity spaces for generating renewable energy...” |
| . L215, Figure 1 and Table 1. The word “consistent” alone does not explicitly explain the meaning that I think you are applying to this term. If I understand it correctly, you do two comparisons: an aspatial comparison of total areas mapped as a certain class, and a spatial overlay operation where you look for agreement in class labels of pixels. You appear to use “consistent” to mean the latter only. I think to make this usage clearer it would help a lot to say “consistent labels” or “consistent labeling” or “consistency in labeling of pixels” or “spatial agreement”. I also think you should state in the methods that you have these two ways of evaluating agreement. | Thank you for this comment. We added an explanatory sentence to the method section to specify what is meant with consistent in this case.It reads as follows:  “We compare resulting maps also in terms of spatial consistency, meaning areas where there is a spatial match of the class labels between the different outputs.” |
| 9. L221 and also figure 2. In some cases you use the term “area” where I think you mean “class” or “label”. For example, in L221 – I think the point you are making is that errors in the delineation of certain classes can have a large effect on the model outcome. | We understand the confusion and have rewritten the sentence as follows:  “… highlight the importance of a good representation of sites of importance for climate protection. “ |
| 10. L227 – “this case” – do you mean “this class”? I’m also confused about “deficits” and “untapped” – it would be clearer to simply talk about “disagreements” and “differences” or “not mapped”. | Sorry for the confusion, we have revised the sentence for clarity but did not change the exact wording as it would alter the intended message.It now reads:  Surprisingly, using coarser CLC 25 data leads to better results, with a smaller area deficit of around 1,472 hectares that accounts for around 2.8 million t CO_2_ retention potential untapped. |
| 11. L231 – perhaps rephrase along the lines of “In contrast, the use of ATKIS data results in an underestimation of about 5.8 million tons of potential C02 emissions compared to the reference data.” | Thank you, we have rephrased the section. |
| 12. In the next sentence replace with something like “This is because a total of 3,025 fewer ha were mapped as Level 1…” | Thank you, we have rephrased as suggested. |
| 13. Table 2. This is a tricky table to read and absorb. I think it would help a lot to eliminate the use of decimals for the t and ha values. This level of precision (1/100 of 1 ha!) is of little purpose when the numbers differ as much as they do (thousands to millions of ha). Important: the “Potential CO2 emission or retention” cell label for the reference Biotope needs units (presumably t). | Yes, you are absolutely right. We have implemented both changes as suggested. |
| 14. L248 – add a caveat “if we assume the Biotope mapping is correct.” | We have added a more detailed explanation in the data description section where we state the following:  “…as it is the most detailed type of land-use data used for environmental planning in Germany and we assume that it depicts the real circumstances best.” |
| 15. L251 – It wouldn’t be potentially disregarded, but would be disregarded – i.e. rephrase something like “This would lead to a total of 255 ha of areas important to conservation not recognized in the planning recommendations due to the lower detail of the data used.” | Yes, we have done as suggested. Thanks so much for the very useful recommendations. |
| 16. Table 3 – indicate in the table that Biotope is the reference mapping | Thanks, we have done accordingly. |
| 17. Table 3 – need to clarify in the headings that it is “class label” that matches, or that the “class is not mapped” – rather than the area that is missed or consistent (which would mean something else). | Thank you. We have added a title for column 2 to clarify what is meant. The column title reads”Type of area considered”. We also added some specification in the cells below where needed for further clarification. |
| 18. L302 – thematic attributes - do you mean fewer classes? | Yes, this is what we mean. We have corrected it accordingly. |
| 19. L307- thematic information is lacking – do you mean that if the thematic classes do not match the classes of interest? | Yes. We revised the sentence to be more comprehensible. |
| 20. L312 – 2980 km2 – the significance of the area is hard to understand – I recommend also specifying this as a percentage. | Thanks for the important comment, we have integrated the following:  “Thus, the potential area for onshore wind energy in the second model run is 34% smaller than in the first model run.” |
| 21. L323- easy data – do you mean “easily obtained data?? | We changed to easily obtained data. |
| 22. L344 – albeit this would not be necessary – do you mean “that are not actually helpful based on the real land use of the area.” | Yes, this is what we mean. We changed the sentence as suggested. |
| 23. L347 – I don’t understand what this fourth constellation is – do you mean something like “when the potential effort or response required to address the issues raised by the model outcome is large but the data uncertainty is also large? | Yes, this is what we mean.  We now write, as suggested:  “… the fourth problem constellation is when the potential effort or response required to address the issues raised by the model outcome is large but the data uncertainty is also large. This applies, for example, even if the effect would be harmless, as… “ |
| 24. L375-6 – I can’t understand this sentence. | Thank you for pointing this out. Indeed, this sentence is distorted. We have revised it and now write  “The realistic dilemma of using easily available data instead of better but hard to obtain data with uncertain added value in terms of relevance for answering the planning question will not vanish in the near future.” |
| 25. L394 – I don’t understand the sentence (“in how far”?) and what does “behavioral mechanisms” mean? Can you rephrase this? | Thank you for flagging this. We have revised it as follows:  “Finally, in order to enhance the acceptability of uncertainty communication to decision makers, future research should explore how behavioral interventions such as splitting complex, uncertain decisions into smaller decisions with regard to space and time (29) would increase acceptability.” |
